# Supplementary material for: Metabolomic Profiling of Antioxidant Compounds in Five Vachellia Species
Source: Molecules. 2021 Oct 14;26(20):6214. doi: 10.3390/molecules26206214 (PMC8539452; doi:10.3390/molecules26206214)
Supplement: Supplementary file 1 [file molecules-26-06214-s001.zip › molecules-1371964-supplementary.pdf]

molecules

MDPI

Supplementary material

Metabolomic Profiling of Antioxidant Compounds in Five *Vachellia* Species

Garland Kgosi More [1,*], Stephen Meddows-Taylor [2] and Gerhard Prinsloo [3]

[1] College of Agriculture and Environmental Sciences Laboratories, University of South Africa, Florida, Johannesburg, 1710, South Africa
[2] Department of Life and Consumer Sciences, College of Agriculture and Environmental Sciences, University of South Africa, Florida, Johannesburg, 1710, South Africa; mtayls@unisa.ac.za
[3] Department of Agriculture and Animal Health, College of Agriculture and Environmental Sciences, University of South Africa, Florida, Johannesburg, 1710, South Africa; prinsg@unisa.ac.za
* Correspondence: moregk@unisa.ac.za

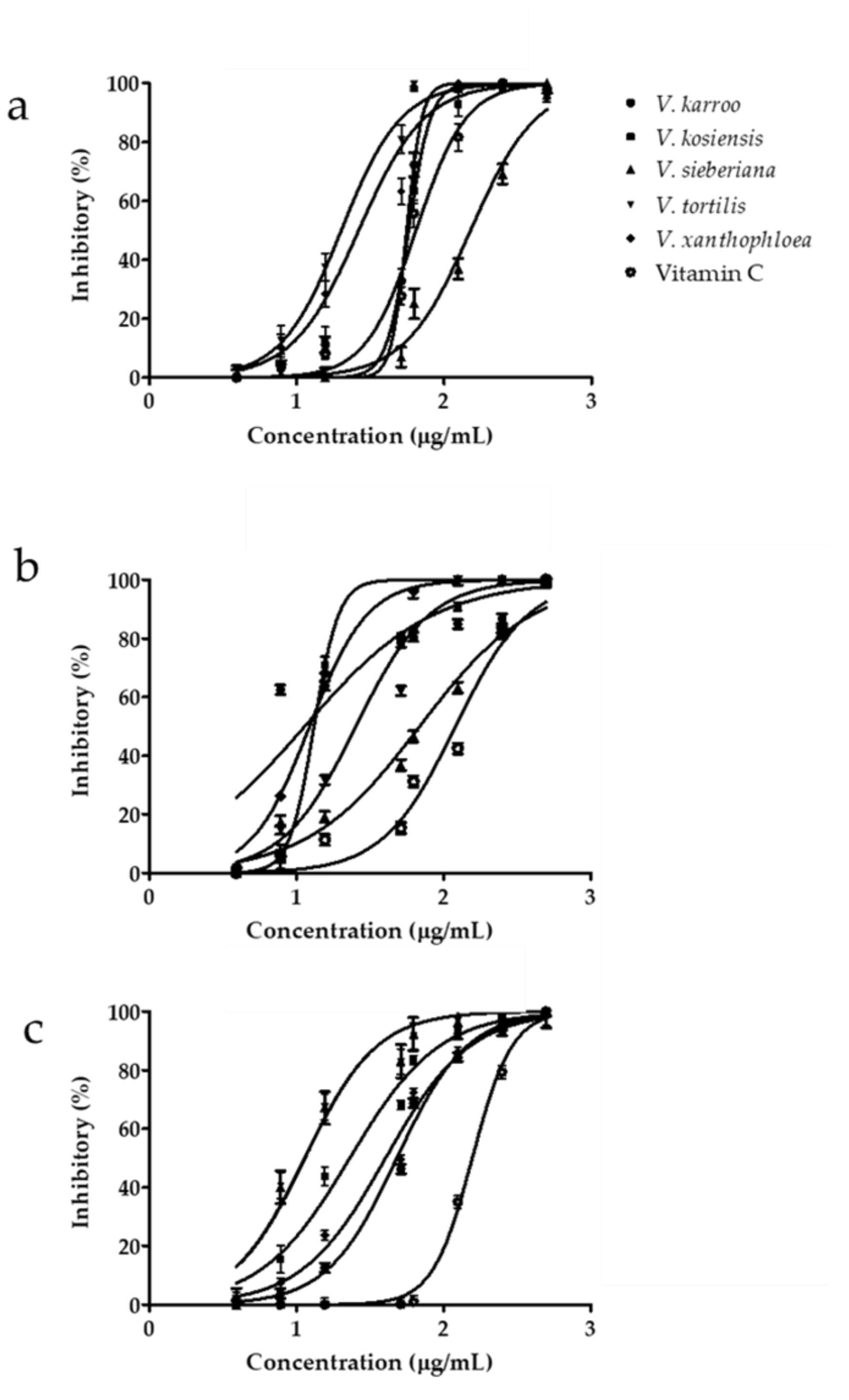

Figure S1. Sigmoidal curved graphs of concentration response of five *Vachellia* extracts against DPPH **(a)**, ABTS **(b)** and FRAP **(c)** with R^2 coefficient ≥ 0.9.

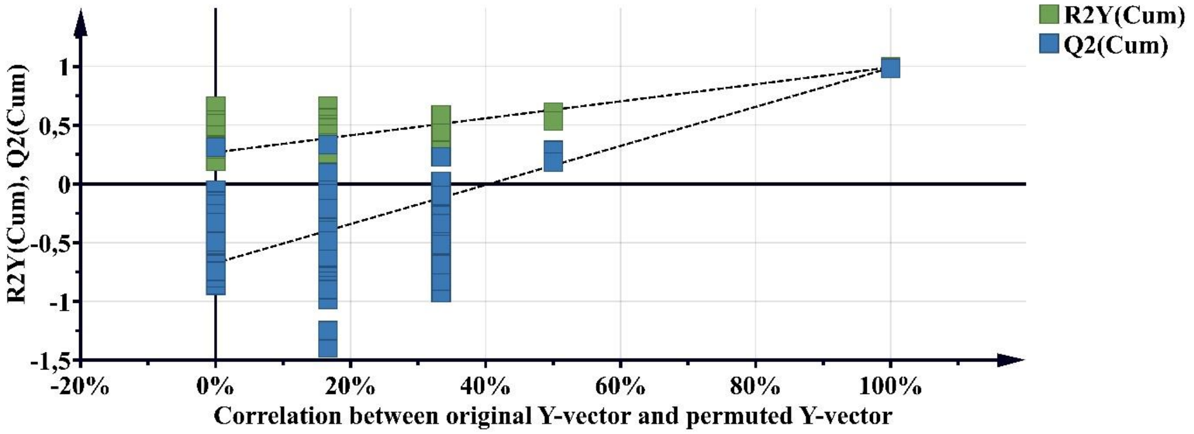

Figure S2. The OPLS-DA derived validation plot (permutation times n = 100) of *Vachellia* samples showing model intercepts of Intercepts: R2 = (0.0, 0,269), Q2 = (0.0, −0,674).

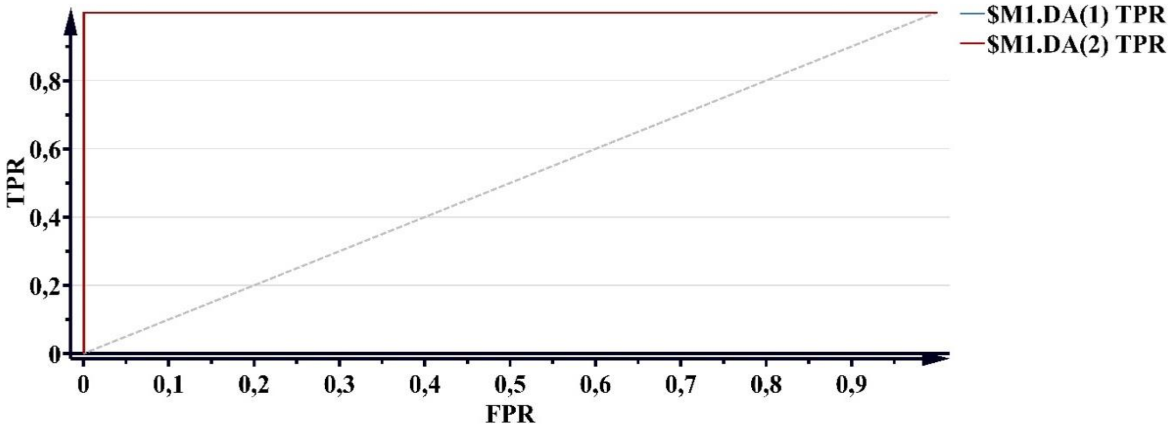

Figure S3. A receiver operating characteristic curve (ROC) area under a curve (AUC) plot with predictably accuracy values of 0.994 and 0.945.

Table S1. Cross validated ANOVA (CV ANOVA) with a p value (1,23 × e-16) of CV ANOVA.

M1(Untitled)	SS	DF	MS	F	p	SD
Total corr.	24	24	1			1
Regression	23,737	6	3,95616	270,732	1,23003e-16	1,98901
Residual	0,263031	18	0,0146128			0,120884

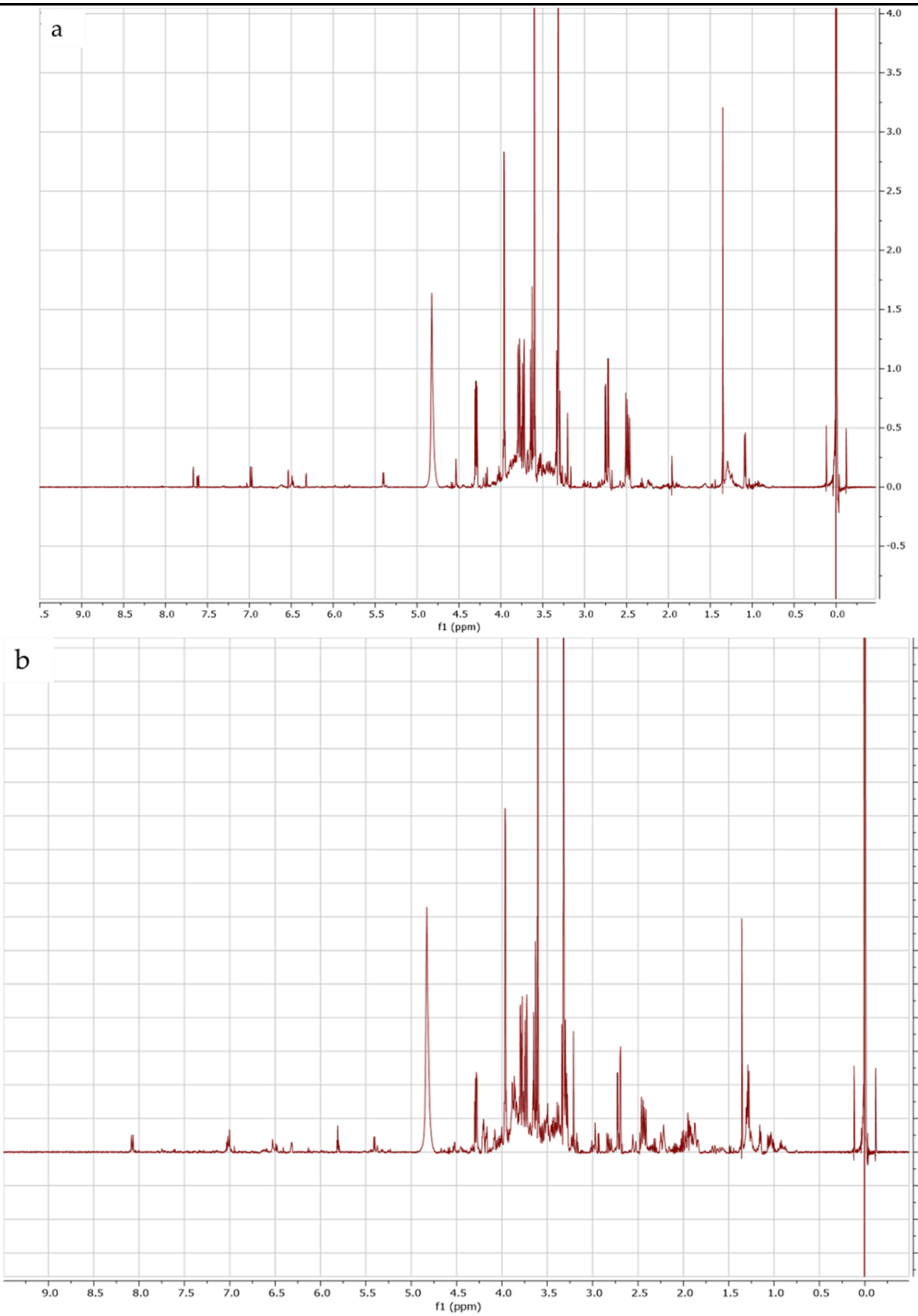
a
f1 (ppm)
b
f1 (ppm)

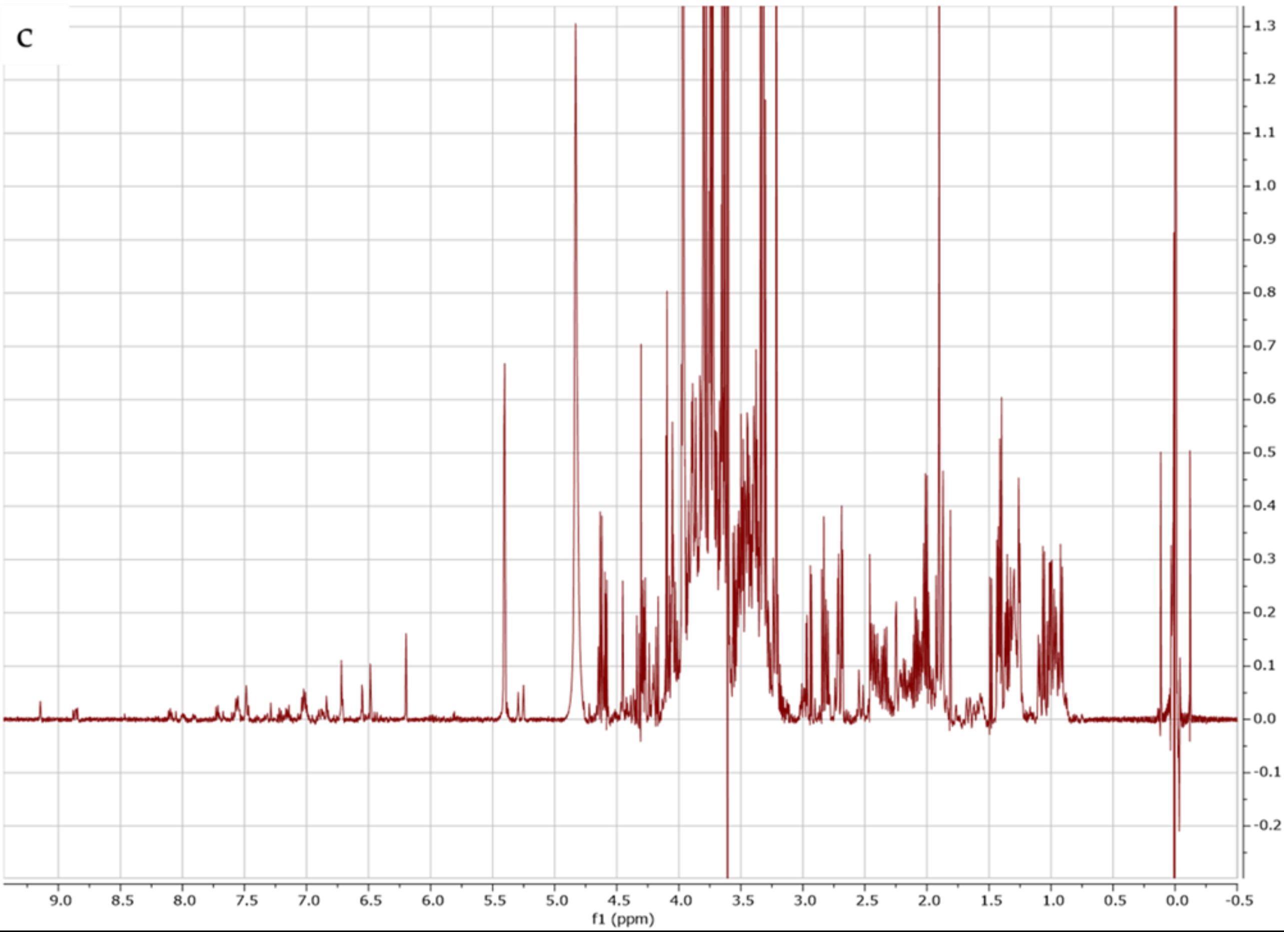
c
9.0
8.5
8.0
7.5
7.0
6.5
6.0
5.5
5.0
4.5
4.0
3.5
3.0
2.5
2.0
1.5
1.0
0.5
0.0
-0.5
f1 (ppm)
1.3
1.2
1.1
1.0
0.9
0.8
0.7
0.6
0.5
0.4
0.3
0.2
0.1
0.0
-0.1
-0.2

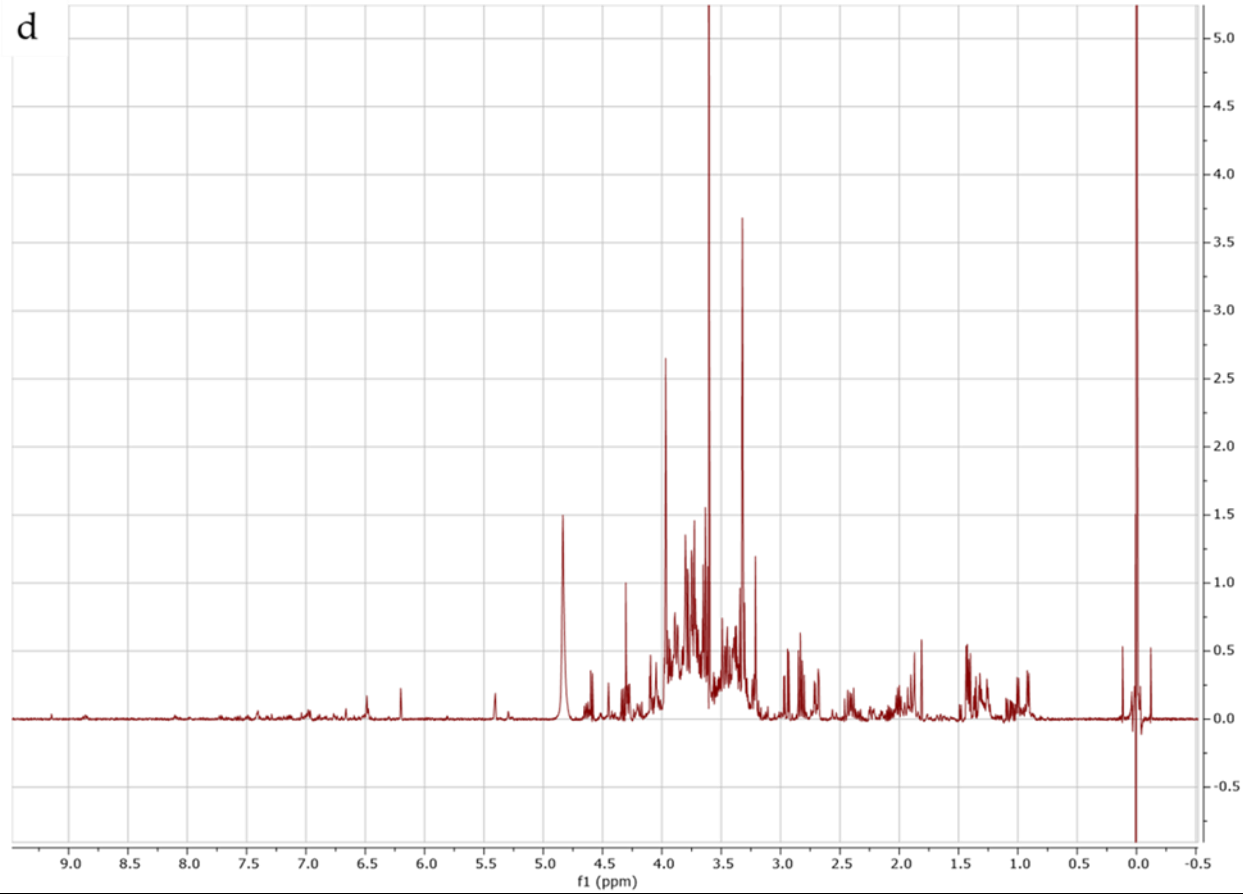
d
9.0
8.5
8.0
7.5
7.0
6.5
6.0
5.5
5.0
4.5
4.0
3.5
3.0
2.5
2.0
1.5
1.0
0.5
0.0
-0.5
f1 (ppm)
5.0
4.5
4.0
3.5
3.0
2.5
2.0
1.5
1.0
0.5
0.0
-0.5

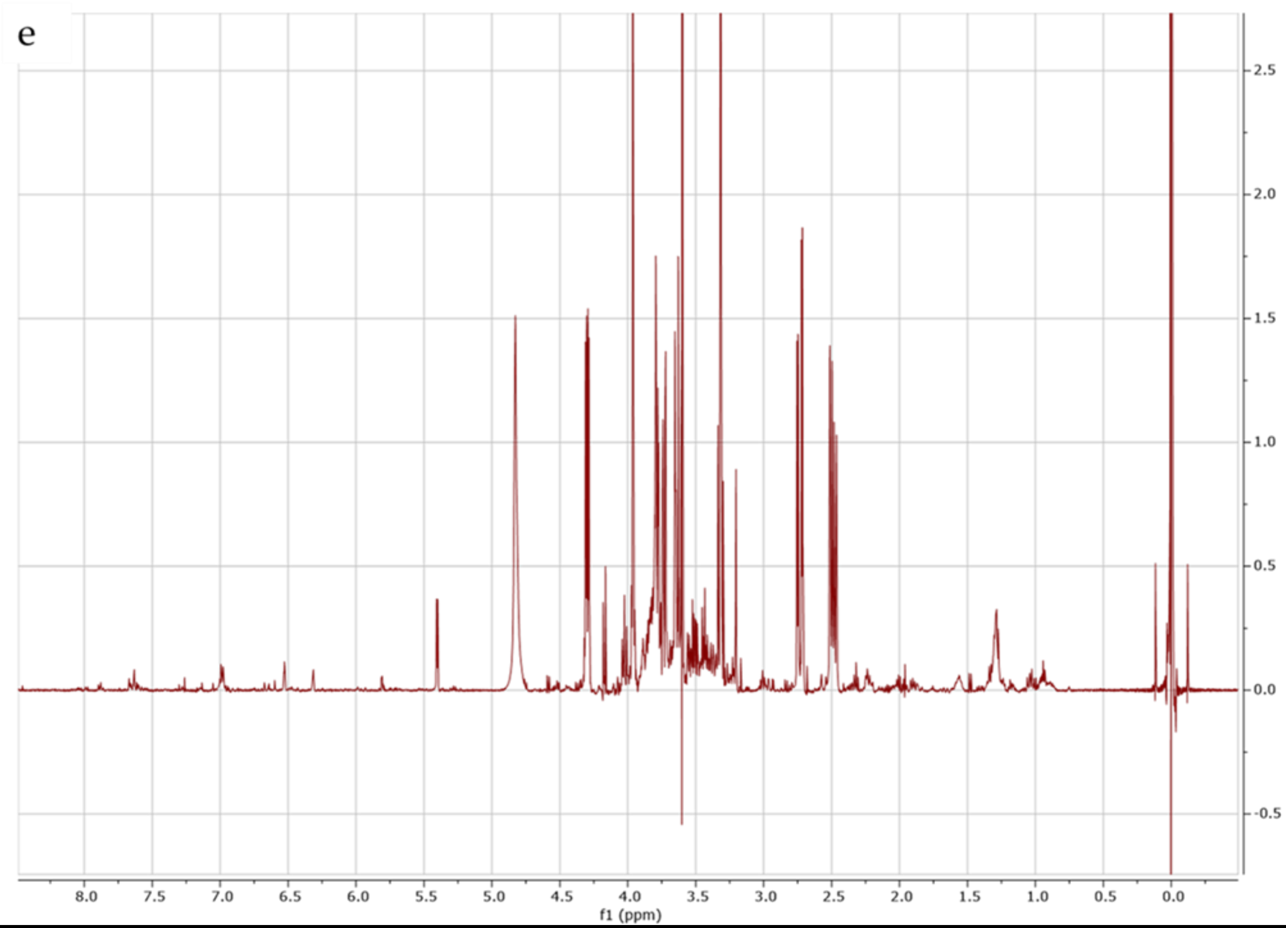

Figure S4. Proton nuclear magnetic resonance (^{1}H NMR) spectrum of 80 % aqueous-methanol plant extract of *Vachellia karroo* **(a)**, *Vachellia kosiensis* **(b)**, *Vachellia sieberiana* **(c)**, *Vachellia tortilis* **(d)** and *Vachellia xanthophloea* **(e)** acquired on a 600 MHz NMR.
